# The Supplementation of Docosahexaenoic Acid-Concentrated Fish Oil Enhances Cognitive Function in Puppies

**DOI:** 10.3390/ani13182938

**Published:** 2023-09-16

**Authors:** Roberta Bueno Ayres Rodrigues, Rafael Vessecchi Amorim Zafalon, Mariana Fragoso Rentas, Larissa Wünsche Risolia, Henrique Tobaro Macedo, Mariana Pamplona Perini, Amanda Maria Gomes da Silva, Pedro Henrique Marchi, Júlio César de Carvalho Balieiro, Wandréa Souza Mendes, Thiago Henrique Annibale Vendramini, Marcio Antonio Brunetto

**Affiliations:** 1 Pet Nutrology Research Center (CEPEN-PET), Department of Animal Nutrition and Production, School of Veterinary Medicine and Animal Science, University of Sao Paulo (USP), Pirassununga 13635-000, Brazil; robertabayres@gmail.com (R.B.A.R.); rafael.zafalon@usp.br (R.V.A.Z.); mariana.rentas@usp.br (M.F.R.); larissa.risolia@usp.br (L.W.R.); henrique.tobaro@hotmail.com (H.T.M.); mariana.perini@usp.br (M.P.P.); amanda-gomes@uol.com.br (A.M.G.d.S.); pedro.henrique.marchi@usp.br (P.H.M.); balieiro@usp.br (J.C.d.C.B.); 2 Avert—Biolab Animal Health, São Paulo 04588-133, Brazil; wmendes@biolabfarma.com.br

**Keywords:** canine, cognition, docosahexaenoic acid, dog, eicosapentaenoic acid, fish oil, learning

## Abstract

**Simple Summary:**

The docosahexaenoic fatty acid, derived from marine sources such as algae and cold-water fish, can be widely incorporated into the brain, especially during the cerebral developmental phase, directly influencing the individual’s cognitive function. This study aimed to evaluate the effects of docosahexaenoic fatty acid supplementation in puppies on cognitive function, through the object discrimination test, which measures the ability of learning. Twelve 3-month-old puppies were used, distributed into two groups of six animals each: the Control Group (CG), without supplementation, and the Experimental Group (EG), with docosahexaenoic acid-concentrated fish oil supplementation. The duration of the study was 3 months; therefore, the animals were evaluated before the beginning (T0) and 30 (T1), 60 (T2) and 90 days (T3) after supplementation. The object discrimination test was composed of two phases, the normal stage and a reverse stage. An effect of supplementation was observed in the normal stage (*p* = 0.0039), so that animals that received docosahexaenoic acid-concentrated fish oil supplementation had a higher frequency of correct answers (70.12%), when compared to puppies that did not receive the supplementation (62.67%). It is concluded that docosahexaenoic fatty acid supplementation was effective and improved the cognitive function of puppies.

**Abstract:**

Docosahexaenoic acid (DHA) has an important role in brain development and functionality. Therefore, this study aimed to evaluate the effects of DHA-concentrated fish oil on the cognitive function of puppies. Twelve 3-month-old puppies were included, blocked by breed and randomly distributed into two groups: the Control Group (CG), without supplementation, and the Experimental Group (EG), supplemented with 40 mg DHA/kg BW/day. The object discrimination test was used, with a normal stage (NS) and a reverse stage (RS), and blood samples were collected to evaluate the serum polyunsaturated fatty acid (PUFA) concentration and total antioxidant capacity (TAC) before (T0) and 30 (T1), 60 (T2) and 90 (T3) days after beginning the study. For the NS, there were effects of treatment (*p* = 0.0039) and time (*p* < 0.0001), in which the correct answer frequency in the EG was higher than the CG. The serum eicosapentaenoic acid (EPA) + HA concentrations at T1, T2 and T3 were higher than at T0 for the EG (*p* = 0.0159), in addition, EG showed higher serum EPA + DHA concentrations than CG at T2 (*p* = 0.0245). The TAC values were similar between the groups (*p* = 0.3211). It was concluded that the cognitive function of puppies can be enhanced with DHA-concentrated fish oil supplementation without increasing the serum lipid oxidation.

## 1. Introduction

Docosahexaenoic acid (DHA) is a polyunsaturated fatty acid (PUFA) of the omega-3 family, considered essential for dogs during the growth phase and for pregnant and lactating females. This fatty acid comprises most of the total fatty acids in brain tissue; therefore, it is considered fundamental in the development and performance of the nervous system, as it can control processes linked to an individual’s learning ability [1,2,3,4]

After birth, the canine brain continues to develop for approximately 60 days and increase in size until the dog reaches adult age [5]. During growth, the brain is more susceptible to the deposition of fatty acids such as DHA, which can be found in higher concentrations in the brain than other fatty acids because it is deposited in a more selective manner [6,7,8,9]. This deposit can be influenced by the diet because the inclusion of DHA in the diet is directly linked to its deposition in the brain with consequent changes in the cognitive function [10,11,12].

Several researchers have developed methodologies that can measure canine cognitive function and investigate how much of it can be influenced by nutritional intervention [13,14,15]. There are studies that evaluated the effects of different nutritional components, such as antioxidants, medium chain triglycerides and DHA in cognitive function of elderly dogs [13,16,17]. However, there are few studies that have investigated the effects of DHA supplementation in dogs during the growth phase [10], in which there is greater incorporation of this fatty acid in the brain tissue, so we believe that in this phase the animals may respond better to supplementation.

Omega-3 PUFAs are characterized by a higher number of double bonds within their molecular structure, which make them more sensitive to oxidation processes. Because docosahexaenoic acid (DHA) easily accumulates in plasma membranes as a component of their structure, an excess of lipid peroxidation can cause various health complications [18].

Given the above, the aim of this study was to evaluate the effects of DHA-concentrated fish oil supplementation on the cognitive function of puppies and to monitor the supplementation effects by evaluating the total antioxidant capacity.

## 2. Material and Methods

All experimental procedures were approved by the Ethics Research Committee for Animal Welfare of the School of Veterinary Medicine and Animal Science at the University of São Paulo (protocol number: 3416110219).

### 2.1. Animals and Experimental Design

The study was conducted at the Pet Nutrology Research Center (CEPEN-Pet) of the School of Veterinary Medicine and Animal Science of the University of São Paulo, Pirassununga, Brazil.

Twelve 3-month-old puppies, six males and six females, were included in the study. These puppies belonged to the Cocker Spaniel breed, with an initial and final average body weight of 8.26 ± 1.21 kg and 13.23 ± 2.25 kg, respectively, and Beagle breeds, with an initial and final average body weight of 7.56 ± 0.60 kg and 11.48 ± 0.71 kg, respectively. All dogs had an average body condition score (BCS) of 5.0 ± 1.0 according to Laflamme [19] and an average muscle mass score (MMS) of 2.0 ± 0.5 as per Michel [20]. The dogs were accommodated in pens measuring 3.42 m^2^ in a covered area, which had an additional outdoor space of 7.21 m^2^, featuring concrete flooring and tiled walls. The puppies were grouped based on predetermined social affinity and provided with ad libitum access to water. Moreover, in order to promote animal welfare, the dogs were given the opportunity for recreation and physical activity twice a day in grassy parks, each with an area of 40 m^2^ per park.

The dogs were provided with complete commercial dry food for puppies and complete commercial wet food for adult dogs as a reward during the cognitive function test, the details of which are outlined later. The amount of wet food provided during the cognitive function testing was calculated to correspond to 10% of daily calories. The intake of both foods was recorded daily, and the estimated nutrient intake was calculated based on total food intake. The fecal score according to Moxham [21] was evaluated, and the daily energy requirement for growth was determined according to the equation 130 × BW^0.75^ × 3.2 × (e^(−0.87 *p*)^ − 0.1) [22], in which BW is the current body weight and *p* corresponds to the BW/expected adult BW. The expected adult BW was considered 14 kg for Cocker Spaniels and 11 kg for Beagles.

The dogs were blocked by breed and were distributed in two groups: the Control Group (CG) consisted of two female Beagles and one male Beagle and two female Cocker Spaniels and one male Cocker Spaniel, which were not supplemented; and the Experimental Group (EG) consisted of one female and two male Beagles and one female and two male Cocker Spaniels, which were supplemented with a commercial DHA-concentrated fish oil Babyox^®^, from Avert—Biolab Animal Health, São Paulo, Brazil (EPA:DHA ratio = 1:4.5 (according to the label)). The amount of supplement provided was readjusted according to BW to obtain a dosage of 40 mg DHA/kg BW/day, with a total intake of approximately 67 mg DHA/kg BW/day when considering the total DHA intake (dry food + wet food + supplementation), as DHA is an essential nutrient and must be present in diets for puppies. The supplementation was provided daily at 8 a.m. in capsules of 500 mg of fish oil, which were administered orally by the responsible veterinarian. The animals were weighed weekly, and predictions were made for the cumulative dosage over the specified period, resulting in an average daily consumption rate of 40 mg DHA per kg of body weight. This average consumption rate is justified by the inherent nature of fish oil capsules that cannot be rationalized and each contain 500 mg. As a result, on certain days within the period of the study, individual dogs might have received more or less capsules per day. Blood samples were collected before supplementation (T0) and after 30 (T1), 60 (T2) and 90 days (T3).

### 2.2. Laboratory Analysis

After an overnight fasting of 12 h, blood samples were collected from the jugular vein. To determine the serum PUFA concentrations and total antioxidant capacity (TAC), 2 mL of blood was collected for each analysis and placed in tubes (BD Vacutainer^®^, Becton Dickinson, New Jersey, United States), containing clot activator. Samples were kept at room temperature for approximately 2 h and were then centrifuged for 15 min at 3500 rpm at 4 °C and the supernatant serum was transferred to 2 mL plastic tubes that were stored at −20 °C.

For the analysis, the serum was thawed, and the fatty acids were identified and quantified by gas chromatography–mass spectrometry (GC-MS) (CG-14B, Shimadzu) at the Laboratory of Biochemistry and Molecular Biology of the School of Agricultural and Veterinary Sciences of the Sao Paulo State University (Júlio de Mesquita Filho), Jaboticabal-Brazil.

The TAC was determined by the Antioxidant Assay (DTAC-100) kit (BioAssay Systems), which measures the TAC in which Cu^2+^ is reduced to Cu^+^ by the antioxidants present in the sample. The resultant Cu^+^ creates a colored complex with the reagent and the color intensity at 570 nm is proportional to the TAC of the sample. The analyses were carried out at the Laboratory Specialized in Scientific Analysis, São Paulo-Brazil.

Regarding food analyses, the dry food samples were ground in a Willye knife mill (Marconi MA340, Piracicaba, Brazil) (sieve of 1 mm). The wet food samples were previously dehydrated in a forced circulation oven at 55 °C for 72 h and later were ground in an analytical mill (Ika, A11 Basic Mill, Staufen, Germany). Dry matter was determined in an oven at 105 °C (Fanen 315, São Paulo, Brazil) according to AOAC [23]. Crude protein (CP) analysis was performed by the Kjeldahl method [23]. The crude fat analysis was performed by the Soxhlet method after acid hydrolysis, and ash was determined using a muffle furnace at 550 °C [23]. The crude fiber was determined according to the Weende method [24]. The analyses were performed in duplicate, and repetition was performed when the coefficient of variation was greater than 5.0%. For the analyses of calcium and phosphorus concentrations, the dry mineral solution was initially prepared, as described by the AOAC [25]. The phosphorus was determined by the colorimetric method, and for the calcium analyses, the EDTA titulometry method was used [25]. The analyses were performed at the Multiuser Laboratory of Animal Nutrition and Bromatology of the School of Veterinary Medicine and Animal Science of the University of São Paulo—Pirassununga, Brazil.

Fatty acid analyses in food samples and fish oil capsules were performed by gas chromatography–mass spectrometry (GC-MS) (CG-14B, Shimadzu, Kyoto, Japan) at the Laboratory of Biochemistry and Molecular Biology of the School of Agricultural and Veterinary Sciences of the Sao Paulo State University (Júlio de Mesquita Filho), Jaboticabal-Brazil.

### 2.3. Learning Capability Evaluation

The learning capability was assessed by an object discrimination test using the Toronto General Testing Apparatus (TGTA) adapted from previous studies [10,14,26]. The test consisted in the ability of the animal to associate an object to a reward and, therefore, measures the learning capability.

The first evaluator (Evaluator 1) was separated from the dog by a curtain so that there was no visual contact between them, and so he could provide the reward. At the opposite side, there was a second evaluator (Evaluator 2) that would put the animal into position inside the apparatus so that he could walk towards the tray. Both evaluators had no knowledge if the animal was in the CG or in the EG. The reward consisted in a complete wet food for puppies (120 g/day of testing) to keep the animals motivated due to its high palatability.

This test consisted of two phases: training and assessment. The training phase comprised four stages:

(a) Adaptation: With the aim of making the animal comfortable in the apparatus and teaching them to walk through it towards the reward tray, every time the animal reached the opening with the adjustable bars, they were rewarded. This procedure was repeated until there was no demonstration of fear of the apparatus (barking, retreating, running).
(b) Reward approach: The aim of this stage was to ensure the association between the tray with feeding. The reward was placed in one of the openings and the animals were conditioned to look for the food. A total of 10 attempts per session were executed, twice a day, and when the animal achieved a reward search in 9 out of 10 in one session or 8 out of 10 in two consecutive sessions, the animal moved on to the next stage. It was considered an error when the animal searched the opening with the tray that did not contain the reward.
(c) Object dislocation: This stage’s aim was to teach the animal to push an object placed in a random position (after a draw) into one of the tray openings that contained the reward, associating the presence of the object to the reward. To avoid choices influenced by smell, the same food was placed in both openings, but one of them did not allow access to the reward. Sessions were composed of 10 attempts, and the same criteria were used as the previous stage to move on to the next and final stage of training. An error was considered when the animal went toward the opening that did not contain the object and, therefore, did not receive the reward.
(d) Choosing the object: In the last stage of training, an object was placed in each tray opening but only one contained a reward, which was denominated S+ (positive stimulus) and had the same shape and color in every attempt. The object that had no food was denominated S− (negative stimulus) and was represented by five different objects in shape and color. The positioning of the objects was altered so that S+ and S− would be present five times on the right and five times on the left, but not more than two times in a row. The same criteria as the last stage were used to complete training.

The duration (days) of each stage of training varied according to each animal. After finishing the training stage, the assessment stage began and went on for 10 days (meaning 10 sessions, 1 per day). Each session had 20 attempts, in which two different shaped objects (one blue and one orange) were used. The objects were randomly assigned between sessions so that they were positioned 10 times on the left and 10 times on the right, but not more than 2 times in a row.

During the first five days of assessment, the normal stage was conducted, in which the orange object was the S+ and the blue object was the S−. In the last five days, the objects were switched, and the blue became S+ and the orange S−, which was considered as the reverse stage. An error was considered when the S− was chosen by the dog. The assessment stage was carried out during the times T0, T1, T2 and T3.

### 2.4. Statistical Analysis

The Statistical Analysis System (SAS 9.4) was used. The residue adherence to normality was assessed by the Shapiro–Wilk test and variables that did not comply with the statistical premises were log transformed or r-squared and, when necessary, outliers were removed. The nutrient intake was assessed by variance analysis by PROC GLM, and the variables of the object discrimination test, PUFA serum concentrations and TAC were assessed with the variance analysis by PROC MIXED, which was used with repeated measures in time, and when an effect was identified, the means were compared by the Tukey test, so that *p* ≤ 0.05 was considered significant.

## 3. Results

The results of the bromatological analyses and polyunsaturated fatty acid profile in commercial diets and the fish oil supplement are shown in Table 1.

The EPA + DHA intake was different between the groups (*p* = 0.0001) (Table 2). The CG ingested a mean of 64.77 ± 0.60 mg/kg (57.4% EPA and 42.6% DHA) (Table 2). The EG ingested a mean of 112.22 ± 4.70 mg/kg (40.4% EPA and 59.6% DHA) (Table 2), which represented an increase of 1.7 times the CG. The EPA:DHA ratio provided by the supplementation was 1:3.97 (12.5% EPA and 49.6% DHA) (Table 1). The intake of other nutrients did not differ between the groups (*p* > 0.05) (Table 2).

### 3.1. Learning Capability Evaluation

As shown in Table 3, the correct answers obtained at the normal stage have differed between the groups (*p* = 0.0039) and over time (*p* < 0.0001), for which the mean right responses of the EG (70.12%) was higher than the CG (62.67%), independent of time (*p* = 0.1134). Regarding the reverse stage, there was a time effect (*p* < 0.0001), but there was no effect of treatment (*p* = 0.8516).

To better understand the data, Table 4 shows the mean frequencies of the correct responses during the normal and reverse stages.

### 3.2. Serum PUFA Concentrations

There was an interaction between time and treatment (*p* = 0.0159) in EPA+DHA concentrations (Table 5). The EG showed an increase in serum EPA + DHA concentrations after 30 days of supplementation (T1), then EPA + DHA serum concentrations were maintained at times T2 and T3 (Table 6). In the CG, there was no variation in EPA + DHA serum concentrations over time (Table 6). Regarding the comparison between the groups over time, the EG presented higher EPA+DHA concentrations than the CG at T2 (Table 5).

The values for AA and LA serum concentrations were reduced in both groups under the effect of time (*p* = 0.0158 and *p* < 0.0001, respectively) whilst ALA serum concentrations did not differ between groups and over time (Table 5).

### 3.3. Total Antioxidant Capacity

There was an effect of time of total antioxidant capacity (*p* = 0.0322) independent of treatment (Table 7).

## 4. Discussion

The main findings of this study were the beneficial effects of DHA-concentrated fish oil supplementation during the TGTA cognitive function test normal stage, which assessed the animals’ learning capacity and the increase in serum EPA + DHA concentrations in EG puppies from 30 days after supplementation, without changes in total antioxidant capacity.

In another study, Zicker et al. [10] applied four cognitive tests in Beagles before one year of age, including the object discrimination test. The authors did not observe a significant difference when using this test alone in dogs supplemented with DHA; however, when evaluating all four of the cognitive tests, there was a positive effect in puppies supplemented with 0.19% of DHA, which corroborates the findings of our study.

In the study by Zicker et al. [10], the authors provided three different concentrations of DHA to beagle puppies after weaning, in low concentration (<0.01% DHA), moderate concentration (0.095% DHA) and high concentration (0.19% DHA). Comparing the EPA + DHA intake values of the supplemented group in the present study, the amount provided by Zicker et al. (2012) was higher only for the high concentration group (1.2 times higher), with the low and moderate concentration groups being 20.9 and 1.86 times lower, respectively, than the EG group. The groups evaluated by Zicker et al. [10] showed a higher amount of EPA than DHA in the diets provided to the groups that received high and moderate DHA, but it is not possible to obtain this information from the group that received low DHA, as there is not very precise information for this statement.

Regarding the reverse stage, the data showed an effect of time regardless of treatment (Table 3), that is, both groups showed an increase in correct answers throughout the experiment. The absence of the treatment effect in the reverse stage can be attributed to the fact that EG dogs developed higher learning capacity during the normal stage and faced a greater challenge when performing the reverse stage, due to the degree of complexity associated with the exercise. Linked to this, another factor that may have influenced this result is the number of repetitions in the sessions. However, even though the reverse step was more difficult than the normal one, both groups understood the proposed activity and responded correctly throughout the periods, which indicates that repetition learning is possible even if it took longer for the CG dogs. This is also corroborated by Zicker et al. [10], who in the reference point test observed fewer errors in the last stage than the two previous stages.

Positive effects of DHA supplementation have also been observed in humans during the growth phase. Richardson et al. [27] evaluated the supplementation of 600 mg DHA/day via capsule to children between 7 and 9 years old with low school performance for 16 weeks. It was observed that the supplemented group performed better at reading and had reduced behavior problems. In the study by Henriksen et al. [28], they evaluated the cognitive function and recognition memory of children at 6 months of age, born prematurely and supplemented with 120 mg of DHA and 60 mg of arachidonic acid per day. The authors observed improvement in problem solving ability and recognition memory with supplementation.

The positive effects of DHA on cognitive function occur through different mechanisms; DHA has a modulating effect on the phospholipid synthesis, mainly phosphatidylethanolamine, which influences neuronal survival [29]. In addition to the accumulation and alteration in phospholipids’ composition, the higher intake of DHA can lead to an increase in the synapses’ mediators, beneficial effects on neurite growth and is responsible for the development of neurons, in addition to promoting synaptogenesis and synaptic expression [30,31,32,33]. The enhanced cognitive function obtained in the present study can be explained by the possible esterification of DHA in the cerebral phospholipid membrane, because DHA has a high deposition in the brain and its supplementation may be important to development and function of the nervous system, controlling learning processes [1,2,3,8,9]

DHA deposition in the brain tissue of puppies has been studied by Dahms et al. [11], in which 7-month-old Beagle dogs were divided into five groups, each of them receiving different dosages of DHA for 8 weeks (0, 100, 500, 1000 and 2000 mg/kg BW), and five more groups for 9 months (0, 150, 1000 and 2000 mg/kg BW). Short- and long-term supplementation can increase cerebral deposition of DHA according to those authors, and dosages of 500 mg/kg/day BW for 8 weeks or 150 mg/BW/day in the long term were sufficient to achieve the highest DHA levels in the brain and can remain that way even after 2 months without supplementation.

Canine neonatal cerebral development occurs mainly in the first 60 days of life and goes on until reaching adult age [5]. During growth, there is a high fatty acid deposition in the brain. There are no studies investigating the effect of supplementation in the canine brain up to 6 months of age, but there is evidence of DHA deposition in the brain of rats [7] and humans [34]. The supplemented amount used in the present study may have been sufficient to enhance cognition due to an efficient deposition in the brain; however, it is not possible to confirm this because this analysis was not performed.

Regarding the serum PUFA concentrations, the EG presented higher EPA + DHA concentrations than the CG at T2 (Table 6), which can be explained by the difference in the intake of these nutrients, which was also observed by Zicker et al. [10]. It was expected that the difference in serum EPA + DHA concentrations between the groups would remain until the final of the study (T3), because the supplement amount provided was readjusted over time, according to the animals’ weight gain; in addition, the serum EPA + DHA concentrations did not differ between T2 and T3 in any of the groups (Table 6). The reason why the differences between the groups were not maintained at T3 is not clear.

The serum AA and LA concentrations decreased over time in both groups, regardless of treatment (Table 5). It has already been demonstrated that the dietary omega-3 PUFAs’ addition can decrease the omega-6 fatty acid concentrations in the plasmatic membranes due to competition of substrates and enzyme inhibition [11] and in the plasma [35]. However, it is worth mentioning that in our study, no treatment effect was observed for serum LA and AA concentrations, only a time effect, so it was not clear why there was no difference between groups; perhaps the omega-3 PUFAs amounts used were not enough.

The TAC was assessed to monitor the fish oil supplementation effects in lipid oxidation. The omega-3 PUFAs can be sensitive to the oxidation process due to their number of double bonds [36]. Wander et al. [37] observed that low omega-6:omega-3 ratios in dogs, such as 1.4:1, increased plasma lipid oxidation metabolite concentrations. However, in the present study, the TAC was similar between the groups (Table 7), which suggests that in the dosage used, the higher EPA and DHA intake did not increase oxidation processes in the puppies. These results can be justified by the omega-6:omega-3 intake ratio in our study. The EG had an average omega-6:omega-3 intake of 5.68:1, which despite being lower than the CG (7.12:1), was much lower than that observed in the study by Wander et al. [37].

## 5. Conclusions

It is concluded that DHA-concentrated fish oil supplementation in puppies can positively influence the learning ability, with enhanced cognitive function as evaluated by the object discrimination test. Furthermore, the DHA supplementation promoted an increase in serum EPA + DHA concentrations without causing a change in total antioxidant capacity.

## Figures and Tables

**Table 1:** Results of the bromatological analyses and polyunsaturated fatty acid profile (on a dry matter basis) of the commercial diets and fish oil supplement used in the present study.

Item	Complete Diets			
	Dry Food	CWD	BWD	Fish Oil
Dry matter (%)	87.45	94.16	93.48	-
Ash (%)	6.62	10.74	9.24	-
Crude fat (%)	21.50	23.53	26.77	-
Crude fiber (%)	5.18	11.90	8.21	-
Calcium (%)	1.55	2.04	1.55	-
Phosphorus (%)	0.89	1.91	1.55	-
Crude protein (%)	34.08	48.77	49.21	-
Linoleic acid (%)	0.24	0.43	0.41	1.38
Alpha-linolenic acid (%)	4.19	5.95	5.38	9.00
DHA (%)	0.11	0.02	0.04	49.60
EPA (%)	0.15	0.01	0.02	12.50
AA (%)	0.12	0.37	0.41	1.40

Legend: CWD = Chicken-based wet diet; BWD = beef-based wet diet; DHA = docosahexaenoic acid; EPA = eicosapentaenoic acid; AA = arachidonic acid.

**Table 2:** Mean daily intake of dry matter and nutrients (unit/kg^0.75^) by dogs supplemented and not supplemented with DHA-concentrated fish oil.

Item (unit/kg^0.75^)	CG	EG	SEM	*p* Value
Dry matter (g)	43.85	42.41	2.08	0.6351
Crude protein (g)	18.25	17.32	0.82	0.4435
Crude fat (g)	11.00	10.53	0.50	0.5268
Crude fiber	3.00	3.30	0.36	0.5579
Ash (g)	3.19	3.00	0.15	0.6636
Calcium (g)	0.79	0.75	0.03	0.4306
Phosphorus (g)	0.51	0.49	0.02	0.5079
Alpha-linolenic acid (mg)	91.15	99.87	7.53	0.4325
EPA + DHA (mg)	114.50	200.00	5.48	<0.001
Linoleic acid (mg)	1397.20	1653.40	206.20	0.4001
AA (mg)	59.53	51.43	5.87	0.3526

Legend: CG = control group; EG = experimental group, DHA = docosahexaenoic acid; EPA = eicosapentaenoic acid; AA = arachidonic acid, SEM = standard error of mean.

**Table 3:** Results of the variance analyses (ANOVAs) of correct response frequency in DHA-supplemented and non-supplemented puppies at the normal and reverse stages of object discrimination test.

Variables (%)	Groups		SEM	*p*-Values		
	CG	EG		Treatment	Time	Time × Treatment
Normal stage	62.67	70.12	2.98	0.0039	<0.0001	0.1134
Reverse stage	42.50	43.12	2.35	0.8516	<0.0001	0.5092

Legend: CG = control group; EG = experimental group; SEM = standard error of mean.

**Table 4:** Mean of correct response frequencies at the normal and reverse stages of object discrimination test in DHA-supplemented and non-supplemented puppies.

Normal Stage			
Time Point	Treatment		
	CG (%)	EG (%)	SEM
T0	82.00 ^A^	85.50 ^A^	2.98
T1	64.16 ^B^	77.33 ^B^	
T2	47.66 ^B^	61.33 ^B^	
T3	56.83 ^B^	56.33 ^B^	
**Reverse Stage**			
**Time Point**	**Treatment**		
	**CG (%)**	**EG (%)**	**SEM**
T0	22.16 ^B^	27.83 ^B^	5.34
T1	45.50 ^A^	39.16 ^A^	
T2	48.83 ^A^	54.16 ^A^	
T3	53.50 ^A^	51.33 ^A^	

Legend: CG = control group; EG = experimental group; SEM = standard error of mean; ^A, B^ Means followed by upper case superscript letters in the same column differed by the Tukey test.

**Table 5:** Results of variance analyses (ANOVAs) of serum EPA + DHA concentrations in DHA-supplemented and non-supplemented puppies.

Variables (%)	Treatment		SEM	*p*-Values		
	CG	EG		Treatment	Time	Time × Treatment
EPA + DHA	4.88	5.60	0.32	0.1439	0.00005	0.0159
EPA	2.04	1.84	0.10	0.4525	<0.0001	0.0066
DHA	3.04	3.56	0.21	0.0984	0.0229	0.0412
Arachidonic acid	22.59	22.05	1.01	0.3604	0.0158	0.6822
Linoleic acid	18.14	17.52	0.30	0.1560	<0.0001	0.5088
Alpha-linoleic acid	0.27	0.31	0.06	0.1727	0.1497	0.7946
Oleic	7.50	7.48	0.31	0.9475	0.0727	0.3533
Stearic	24.21	24.50	0.29	0.4821	0.0068	0.7531
Other fatty acids ^1^	22.28	22.53	0.46	0.7041	0.8855	0.5166

Legend: CG = control group; EG = experimental group; SEM = standard error of mean; DHA = docosahexaenoic acid; EPA = eicosapentaenoic acid. ^1^ Myristic, Pentadecanoic, Palmitic, Palmitoleic, Heptadecanoic, Heptadecenoic, Cis-vaccene, Eicosenoic, Eicosadienoic, Eicosatrienoic (cis—8, 11, 14), Eicosatrienoic (cis—11, 14, 17), Docosatetraenoic (DTA), Docosapentaenoic acid (DPA).

**Table 6:** Mean serum EPA + DHA concentration in relative percentage in DHA-supplemented and non-supplemented puppies.

Time Point	Treatment		SEM
	CG (%)	EG (%)	
EPA + DHA			
T0	4.50 ^Aa^	4.26 ^Ba^	0.40
T1	5.04 ^Aa^	5.99 ^Aa^	
T2	4.80 ^Ab^	6.30 ^Aa^	
T3	5.78 ^Aa^	5.82 ^Aa^	
DHA			
T0	2.99 ^Aa^	2.93 ^Ba^	0.28
T1	3.28 ^Aa^	3.86 ^Aa^	
T2	2.88 ^Ab^	4.04 ^Aa^	
T3	3.02 ^Aa^	3.41 ^ABa^	
EPA			
T0	1.61 ^Aa^	1.33 ^Ba^	0.20
T1	1.76 ^Aa^	2.14 ^Aa^	
T2	1.92 ^Aa^	2.27 ^Aa^	
T3	2.07 ^Aa^	2.41 ^Aa^	

Legend: CG = control group; EG = experimental group; SEM = standard error of mean. ^a, b^ Means followed by lower case superscript letters in the same row differed by the Tukey test; ^A, B^ Means followed by upper case superscript letters in the same column differed by the Tukey test.

**Table 7:** Results of variance analyses (ANOVAs) of total antioxidant capacity in DHA-supplemented and non-supplemented puppies.

Variable	Treatment		SEM	*p*-Values		
	CG	EG		Treatment	Time	Time × Treatment
CAT eq/TROLOX mM	0.361	0.375	9.72	0.3211	0.0322	0.9878

Legend: CG = control group; EG = experimental group; SEM = standard error of mean.

## Data Availability

The data presented in this study are available on request from the corresponding author.
